# Health Benefits of Reducing Sugar-Sweetened Beverage Intake in High Risk Populations of California: Results from the Cardiovascular Disease (CVD) Policy Model

**DOI:** 10.1371/journal.pone.0081723

**Published:** 2013-12-11

**Authors:** Tekeshe A. Mekonnen, Michelle C. Odden, Pamela G. Coxson, David Guzman, James Lightwood, Y. Claire Wang, Kirsten Bibbins-Domingo

**Affiliations:** 1 Department of Medicine, University of California San Francisco, San Francisco, California, United States of America; 2 Division of General Internal Medicine, San Francisco General Hospital, University of California San Francisco, San Francisco, California, United States of America; 3 UCSF Center for Vulnerable Populations at San Francisco General Hospital, San Francisco, California, United States of America; 4 School of Biological and Population Health Sciences, College of Public Health and Human Sciences, Oregon State University, Corvallis, Oregon, United States of America; 5 Department of Clinical Pharmacy, School of Pharmacy, University of California San Francisco, San Francisco, California, United States of America; 6 Health Policy and Management, Mailman School of Public Health, Columbia University, New York, New York, United States of America; 7 Department of Epidemiology and Biostatistics, University of California San Francisco, San Francisco, California, United States of America; Charité University Medicine Berlin, Germany

## Abstract

**Background:**

Consumption of sugar-sweetened beverage (SSB) has risen over the past two decades, with over 10 million Californians drinking one or more SSB per day. High SSB intake is associated with risk of type 2 diabetes, obesity, hypertension, and coronary heart disease (CHD). Reduction of SSB intake and the potential impact on health outcomes in California and among racial, ethnic, and low-income sub-groups has not been quantified.

**Methods:**

We projected the impact of reduced SSB consumption on health outcomes among all Californians and California subpopulations from 2013 to 2022. We used the CVD Policy Model – CA, an established computer simulation of diabetes and heart disease adapted to California. We modeled a reduction in SSB intake by 10–20% as has been projected to result from proposed penny-per-ounce excise tax on SSB and modeled varying effects of this reduction on health parameters including body mass index, blood pressure, and diabetes risk. We projected avoided cases of diabetes and CHD, and associated health care cost savings in 2012 US dollars.

**Results:**

Over the next decade, a 10–20% SSB consumption reduction is projected to result in a 1.8–3.4% decline in the new cases of diabetes and an additional drop of 0.5–1% in incident CHD cases and 0.5–0.9% in total myocardial infarctions. The greatest reductions are expected in African Americans, Mexican Americans, and those with limited income regardless of race and ethnicity. This reduction in SSB consumption is projected to yield $320–620 million in medical cost savings associated with diabetes cases averted and an additional savings of $14–27 million in diabetes-related CHD costs avoided.

**Conclusions:**

A reduction of SSB consumption could yield substantial population health benefits and cost savings for California. In particular, racial, ethnic, and low-income subgroups of California could reap the greatest health benefits.

## Introduction

Sugar-sweetened beverages (SSB) –soda, fruit punches, sports drinks, sweetened tea, and other carbonated or non-carbonated drinks that are sweetened with sugar–are the largest source of added sugar in the US diet today. [1], [2] Data from the National Health And Nutrition Examination Survey (NHANES) suggests that the total daily kilocalories from SSB is much higher for adults in communities of color than their white counterparts. Specifically, calories from SSBs represent 9% of the daily caloric intake among African Americans and 8% among Mexican Americans and 5% among whites. [3] Consumption of SSB is high in California, with over 10 million children and adults in California consuming one or more SSB per day, including 24% of adults (6.4 million), 62% of adolescents (2 million), and 41% children ages 2–11 (2.2 million). [4].

Current evidence suggests that higher consumption of SSB is associated with excess calorie intake, which leads to weight gain [5] and increased risk of obesity. [6] Consumption of SSB may even act synergistically with genetic predisposition to increase the risk of obesity in some individuals. [7] High-fructose corn syrup, the most common sugar used in sodas, may have particularly deleterious effects on the liver, resulting in hepatic insulin resistance and the metabolic syndrome. [8] High consumption of SSB also appears to increase the risk of diabetes, [9], [10] hypertension, and coronary heart disease (CHD) independent of the effects on weight, [11]–[13] with studies suggesting that those who consume one drink or more per day double their risk of diabetes and raise their risk of CHD by 23% compared to those who consumed one SSB drink or less per month. [12], [14], [15] In 2005, adult diabetes prevalence in California was 7.8%, three times the Healthy People 2010 target. [16] From 2001 to 2009, diabetes prevalence rose steadily in California, particularly in minority populations; over this period the prevalence of diabetes increased 50% among Mexican Americans and 17% among African Americans. [17] Heart disease is the leading cause of death among all Californians. [18].

In response to the growing burden of diet-related chronic diseases, a number of strategies have been proposed and implemented to reduce SSB intake on a population level. Such approaches generally fall in three categories –1) *education and information sharing*, including both targeted efforts to describe the health effects of excessive SSB consumption, as well as efforts to provide consumers with accurate information through menu labeling to allow them to make healthier choices on their own, 2) *restriction*, particularly to vulnerable groups like school-age children and including limiting availability of these products within the schools or limiting the ability to market these products directly to children, and 3) *taxation*, including sales taxes assessed at the point of sale and more recently excise taxes levied on the producer. [19] The limitations of many of these approaches in effectively curbing SSB consumption have led to recent more sweeping approaches designed to have a greater effect on consumer behaviors and to reach a broader range of consumers. Recently New York City Board of Health proposed a novel approach of restricting beverage portion sizes to 16 oz. that, though ultimately stuck down, was anticipated to result in reductions in SSB consumption. [20], [21] Taxes that raise the price of SSBs more substantially in order to more effectively curb consumer behaviors - usually excise taxes of one penny per ounce – have been debated in many jurisdictions and have been of interest both for their impact on SSB consumption and also as tools for generating revenue that might be used for other programs related to chronic disease prevention. [22], [23] Ballot measures proposing such taxes were recently defeated in California’s city of Richmond and El Monte. One of the common criticisms of these measures is that communities of color and low income persons will suffer disproportionately from the tax burden of these measures. [24].

In this paper, we examine and project the health and economic benefit of a reduction in SSB intake as might be achieved by an excise tax in California over the next decade, using the CVD Policy Model – CA, an established computer simulation of diabetes and heart disease adapted to California. Because California is an exceptionally diverse state, and racial and ethnic minority communities have the highest rates of diabetes and per capita consumption, we projected the health benefit from reduced SSB intake in Mexican Americans and African Americans, as well as those with limited incomes.

## Methods

### The Cardiovascular (CVD) Policy Model- CA

The CVD Policy Model is a dynamic population-based model of coronary heart disease and stroke in U.S. adults that has been used to forecast trends in cardiovascular disease for over 25 years. [25] Details of the Model have been described previously.[25]–[27] A California version of the CVD Policy Model (CVD Policy Model – CA) was created for this analysis using state-specific inputs with the underlying structure of the national model. We used U.S. Census estimates for the age-specific population projections for California from 2013–2022. We used data on Western region participants in NHANES, years 1999–2008, and from the California Health Interview Survey (CHIS), years 2001–2009, for the distribution of the demographic characteristics and risk factors. [17] We assumed that all other estimates in the California Model (i.e. risk factor coefficients, case-fatality rates, etc.) were the same as for the U.S. Model.

The CVD Policy Model - CA code is written in Fortran 95 and compiled using the Lahey Fortran 95 compiler V7.2 (Lahey Computer Systems, Incline Village, Nevada).

### Intake of Sugar-Sweetened Beverages in California

We used self-reported frequency of daily SSB consumption from the 2005 CHIS database [28] and included data on intake of all carbonated and non-carbonated SSB and fruit-flavored drinks, but did not include diet or 100% juice drinks. We used estimates from a recent systematic review of the price elasticity for SSBs of −0.79 to −1.00. [29] Based on this price elasticity, an excise tax on 12 ounce beverages with a pre-tax price of $1.00 would be expected to raise the price of the beverage by 12% and result in a 9.5% to 12% reduction in consumption of these beverages. Notably, because the excise tax is a fixed price per a fixed unit of volume, the decline in consumption could be expected to be even greater among consumers purchasing larger or less expensive beverages. For example, a 32 ounce beverage with a pre-tax price of $1.00 would increase in price by 32%, and based on the price elasticity this would be projected to result in a 25–30% reduction in consumption. Based on these relationships, we hypothesized that the impact of a penny-per-ounce tax would result in a 10%–20% reduction in SSB consumption. We also modeled the impact of a hypothetical 50% reduction in SSB consumption that might be achieved by taxation and additional education and menu labeling efforts to curb consumption.

### Risk Factors and Costs

**Figure 1:**
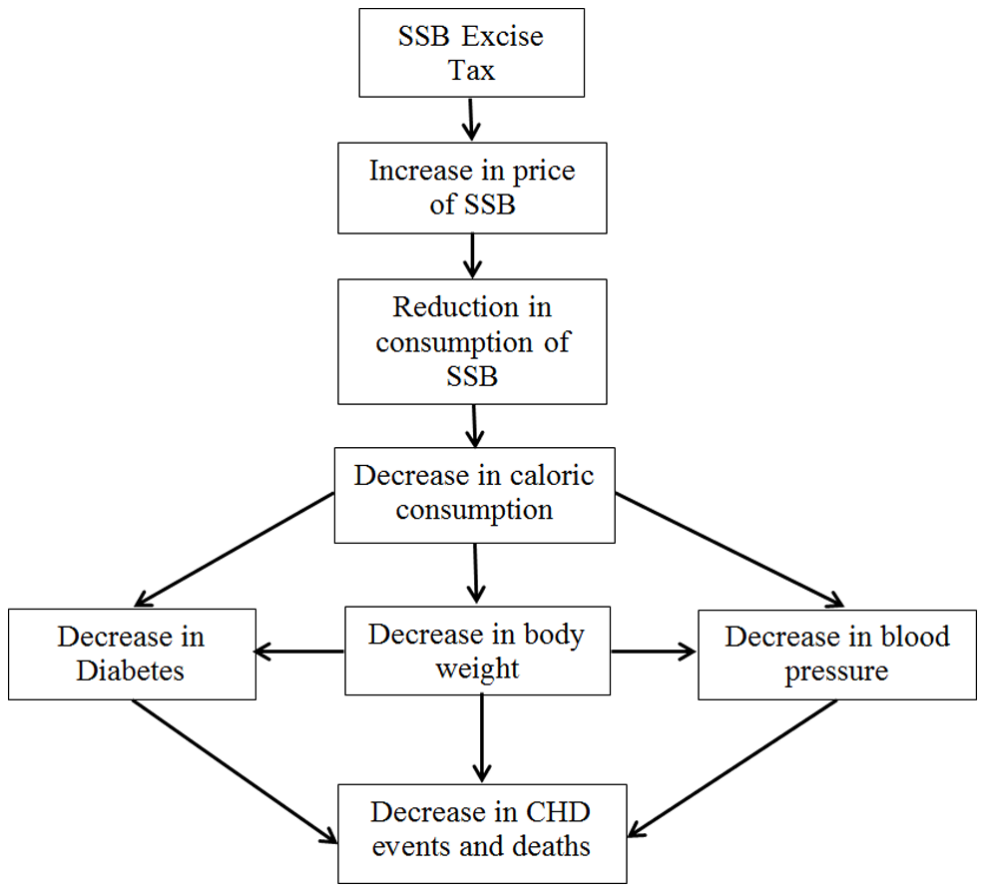
Framework for the impact of an SSB tax on health outcomes.

The difference between the current level of SSB intake and the hypothetical, lower level of SSB intake was translated directly into changes in three cardiovascular risk factors: diabetes, body mass index (BMI), and blood pressure (Figure 1). In addition to these direct effects, lower body weight was assumed to result in additional lowering of blood pressure and diabetes risk. [30] Diabetes and elevated blood pressure were each associated subsequently with an increased risk of CVD events and CVD mortality, and diabetes was associated with additional non-CVD related mortality. The magnitude of the effects modeled at each stage and the associated references are detailed in Table 1.

**Table 1:** Model assumptions.

Risk Factors/inputs	Effect size		Reference
Serving size of a SSB*	12 fl. Oz		
Proportion of calories compensated for by other beverages, after a reduction in SSB	39%		[34], [47]
Relative risk of diabetes associated with consuming one or more SSB per day(95% CI)**	1.35 (95% CI: 1.14, 1.59)		[5]
Proportion of increased risk assumed to be mediated through BMI	50%		[12]
Change per 1 unit increase of (BMI)	Men	Women	[12], [48]–[50]
Systolic blood pressure, (95% CI)***	1.43	1.24	
Cholesterol (mg/dl)***			
Low-density lipoprotein	2.75	2.24	
High-density lipoprotein	−1.55	−0.77	
Diabetes (per unit BMI)	1.26	1.30	
Change in systolic blood pressure due to a reduction in SSB consumption of 1serving/day, mmHg (95% CI)***	−0.78 (95% CI: 0.09, 1.47)	−0.61 (95% CI: −0.27,1.48)	[11]
Change in consumption by elasticity estimate, assuming a pre-tax price of $1.00	−0.79 to −1.00		[29]

Sugar-sweetened beverages.

Hazard ratio.

β coefficients.

To assess the impact of the reduction in SSB consumption on the projected number of new cases of diabetes prevented in California, we used estimates from a published meta-analysis of SSB intake and risk of type II diabetes. [5] Because some, but not all, of the studies adjusted for adiposity and energy intake, we used the estimate for the risk of diabetes associated with each additional 12 oz serving of SSB per day in which energy- and adiposity-adjusted coefficients were excluded (RR = 1.35 (95% CI: 1.14, 1.59). We then adjusted this estimate to account for changes mediated through increased body weight, based on one of the studies included in the meta-analysis. [12].

We estimated the per capita change in calories consumed based on age and sex specific averages of consumption for the state of California. [28] The extent to which reductions in calories from SSB are offset by substituting with other caloric beverages is critical to estimating health impact but also largely unknown. In addition, the relationship between caloric consumption and weight loss is also a topic of debate.[2], [31]–[33] Because of this uncertainty, we varied the impact of a reduction in consumption of SSB on BMI over three scenarios while retaining the independent effects of diabetes and blood pressure:

1. In the most optimistic scenario, we estimated that the entire impact of a decrease in calories due to a reduction in SSB consumption would be translated to weight loss (Strong BMI Effect).
2. In the second scenario, we assumed that 1/3 of the consumption of SSBs reduced due to the proposed tax would be replaced with water, 1/3 with diet drinks, and the final 1/3 with other caloric beverages such as milk and juice. Based on estimates from Stookey et al. of the net impact on daily energy intake from replacing SSB with alternative beverages, [34] we approximated 39% of the SSB calories reduced would be compensated for, resulting in 61% net reduction in daily energy intake (Moderate BMI Effect).
3. In the third scenario, we modeled the extreme scenario that there was no impact of a reduction in SSB on body weight, either due to an adaption of the body to lower caloric consumption or to complete compensation in calories from other food and beverages (No BMI Effect).

Based on the calculation of 3500 kcal/lb, we converted changes in caloric consumption to changes in weight in pounds. We then calculated any corresponding change in BMI for men and women separately, by converting change in pounds to BMI by the formula: BMI = weight (Kg)/height (meters) squared, and using the average height of men and women in the US.

We used an estimate of the direct effect of SSB consumption on systolic blood pressure based on a prospective study of middle-aged men and women. After adjustment for confounders including age, BMI, change in BMI, and physical activity, the authors found that a reduction of SSB consumption by 1 serving per day was associated with a reduction in systolic blood pressure of 0.78 mmHg among men and 0.61 mmHg among women. [11].

The economic costs in this study were estimated from the California’s Office of Statewide Health Planning and Development (OSHPD) and the national Medical Expenditure Panel Survey (MEPS) [35] and only included direct medical costs that are allocated for preventive, diagnostic, and treatment services, costs adjusted to a common national cost basis. We estimated age-specific CHD-related costs (including diabetes costs with co-morbid CHD), as well as age-specific non-CHD related diabetes health care costs. [36] We adjusted the estimated costs to 2012 dollars, based on the Medical Care Consumer Price Index, [37] and costs were discounted 3% annually.

### Simulations

We used the CVD Policy Model – CA to run simulations from the years 2013–2022 to estimate the impact of the SSB consumption reduction. We ran the CVD Policy Model – CA under the baseline scenario and then modeled the impact of the reduction of SSB intake on the distribution of risk factors in order to estimate the subsequent effect on CVD events and mortality. We estimated the preventable cases of incident diabetes, CHD (stable or unstable angina, myocardial infarction, cardiac arrest, stroke, and death), myocardial infarction (initial and recurrent) and all-cause mortality. Our base case simulation projected a 10% reduction in consumption of SSB and we conducted sensitivity analyses assuming a 20% and 50% reduction in consumption of SSB. In addition, we varied the impact of a reduction in consumption of SSB on diabetes, BMI, and blood pressure as a sensitivity analysis. We varied BMI over the three scenarios described above (strong, moderate, and no BMI effect), and independent effects on diabetes and blood pressure over the 95% confidence intervals of the main estimates, without allowing the estimates to be less than zero (a protective effect of SSB consumption on the risk factors). To estimate the impact of the tax in racial and ethnic and low income subgroups in California, we adapted the CVD Policy Model – CA to African Americans, Mexican Americans, and persons with an income less than 200% of the federal poverty line in California. Using the same framework as the CVD Policy Model – CA, we modified the distribution of risk factors to reflect that of the subgroups based on data from NHANES and CHIS for participants whose self-report of race and ethnicity and family income placed them in these categories.

## Results

**Figure 2:**
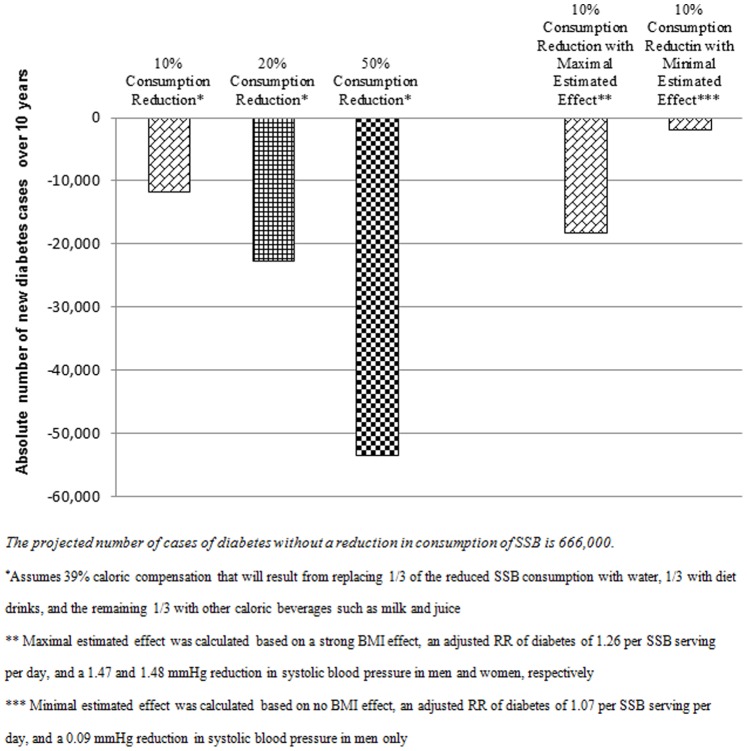
Projected incident diabetes decrease at different levels of SSB consumption reduction with variation of BMI effects.

A reduction in SSB consumption of 10–20% is projected to reduce new cases of diabetes in California considerably. A 10–20% reduction in SSB is projected to lower incident cases of diabetes by 12,000 to 23,000 (a 1.8–3.4% reduction) from 2013–2022. A 50% reduction in consumption in SSB could potentially reduce incident diabetes by 53,000 (8.0%) over the next decade (Figure 2). In addition to the large impact on diabetes, a 10–20% reduction in SSB consumption would have a modest impact on the number of new cases of CHD that are projected to be lowered by 6,000 to 12,000 (0.5–1.0%) (Table 2). We also found a reduction in incident stroke, a small benefit not reported here. Based on sensitivity analyses varying the effect of SSB consumption on diabetes, BMI, and blood pressure over a range of minimum and maximum estimated effect sizes, we project that a 10% reduction in SSB consumption could potential reduce incident diabetes by at least 1,900 cases (a 0.3% reduction) and as much as 18,200 cases (a 3% reduction). We project that a reduction in consumption of SSB of 10% would reduce incident CHD by at least 120 cases (a 0.01% reduction) and as much as 9,700 (a 0.9% reduction), and total MIs by at least 50 (a 0.01% reduction) and as much as 4,400 (a 0.8% reduction) (Table 3).

**Table 2:** Absolute number of coronary heart disease events and deaths prevented from a 10–20% SSB consumption reduction with moderate BMI effects from 2013–2022 in California (Percent change).

	Absolute number of anticipated cases before reduced SSB consumption	10% reduction in SSB consumption*	20% reduction in SSB consumption*	
Incident coronary heart disease (CHD)	1,140,000	−6,000 (−0.5%)	−12,000 (−1.0%)	
Total myocardial infarction (MI)**	560,000	−2,700 (−0.5%)	−5,300 (−0.9%)	
CHD mortality	336,000	−1,300 (−0. 4%)	−2,500 (−0.7%)	
Death from any cause	1,668,000	−1,600 (−0.1%)	−3,200 (−0.2%)	

* Assumes 39% caloric compensation that will result from replacing 1/3 of the reduced SSB consumption with water, 1/3 with diet drinks, and the remaining 1/3 with other caloric beverages such as milk and juice.

** Includes new and recurrent myocardial infarctions.

**Table 3:** Absolute number of events and deaths prevented from a 10% SSB consumption reduction under worst and best case scenarios from 2013–2022 in California (Percent change).

	Absolute number of anticipated cases before reduced SSB consumption	Minimal Estimated Effect* †	Maximal Estimated Effect* ‡
Incident diabetes	666,000	−1,900 (−.29%)	−18,200 (−2.73%)
Incident coronary heart disease (CHD)	1,140,000	−120 (−0.01%)	−9,700 (−0.85%)
Total myocardial infarction (MI)**	560,000	−50 (−0.01%)	−4,400 (−0.79%)
CHD mortality	336,000	−20 (−0.01%)	−2,100 (−0.62%)
Death from any cause	1,667,000	−60 (−0.00%)	−2,700 (−0.16%)

* Assumes a moderate BMI effect of the reduction in SSB consumption: 39% caloric compensation that will result from replacing 1/3 of the reduced SSB consumption with water, 1/3 with diet drinks, and the remaining 1/3 with other caloric beverages such as milk and juice.

** Includes new and recurrent myocardial infarctions.

† Minimal estimated effect was calculated based on no BMI effect, an adjusted RR of diabetes of 1.07 per SSB serving per day, and a 0.09 mmHg reduction in systolic blood pressure in men only.

‡ Maximal estimated effect was calculated based on a strong BMI effect, an adjusted RR of diabetes of 1.26 per SSB serving per day, and a 1.47 and 1.48 mmHg reduction in systolic blood pressure in men and women, respectively.

**Figure 3:**
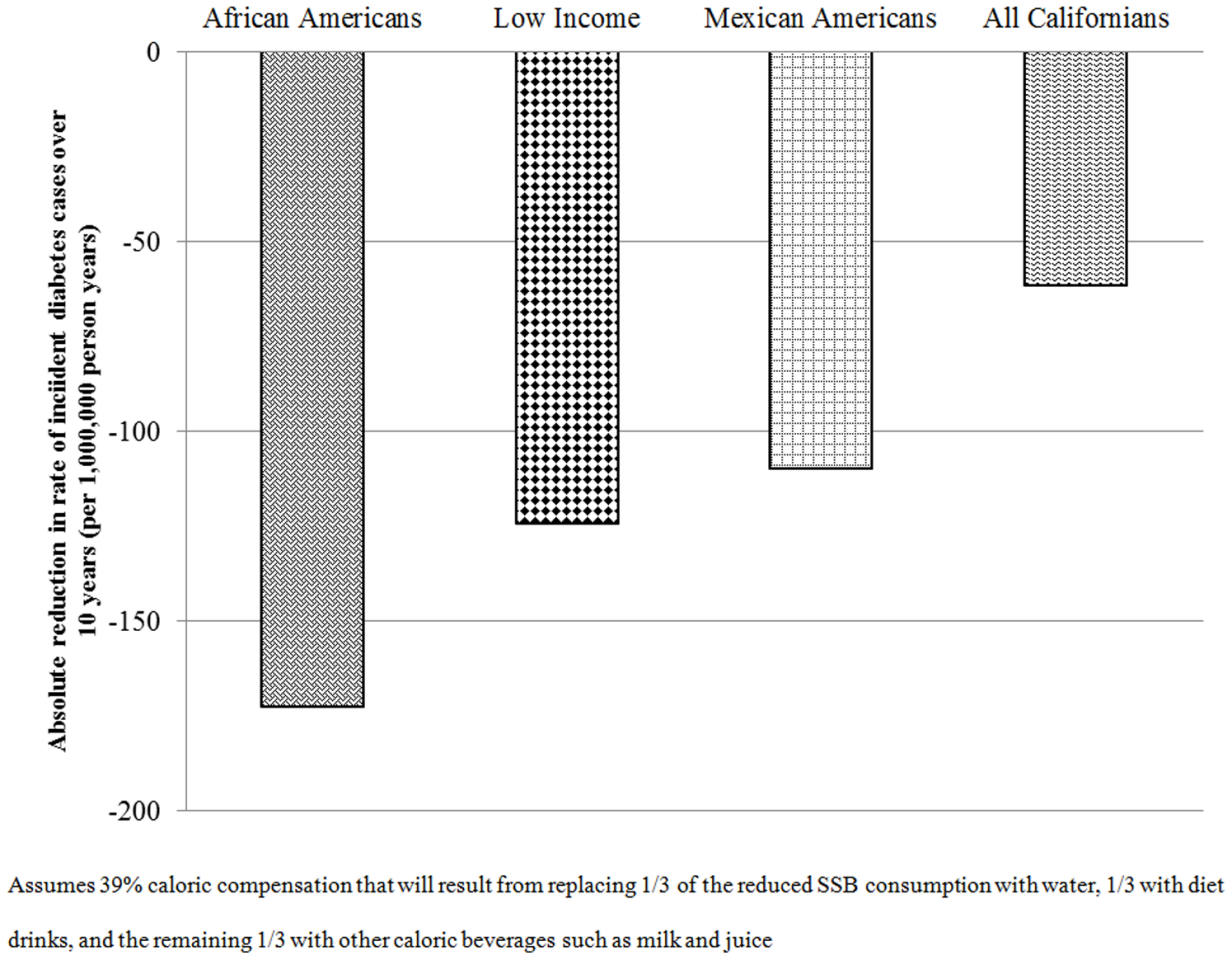
Projected decrease in annual incident diabetes at 10% SSB consumption reduction in subgroups of California.

While all Californians are expected to benefit from reducing SSB intake, the impact of reduction in SSB consumption is projected to have a substantially larger decrease in incident diabetes rates among Mexican Americans and African Americans and those with limited incomes (Figure 3). On average, a 10% reduction in SSB consumption is projected to result in a drop in the rate of new diabetes across California by over 62 per million person-years. For African Americans this rate reduction would triple, dropping by 173 per million person-years, and for Mexican Americans the rate reduction would be expected to be nearly double at 110 per million person-years. Those with limited income, regardless of race and ethnicity, would also be projected to benefit proportionately more than the average effect, with the rate of new diabetes expected to drop by 124 per million person-years (Figure 3). The reductions in rates of incident CHD and all-cause mortality are also projected to be greatest for African Americans, Mexican Americans and those with limited incomes (Table 4).

**Table 4:** Projected difference in event rates per million person-years after a 10% SSB consumption reduction, across subgroups of California (Percent change).

	AllCalifornians*	AfricanAmericans*	MexicanAmericans*	LowSES***
Incident coronary heart disease (CHD)	−35 (−0.54%)	−56 (−0.64%)	−73 (−0.98%)	−53 (−76%)
Total myocardial infarction (MI)***	−17 (−0.52%)	−41 (−0.87%)	−33 (−0.93%)	−27 (−0.77%)
CHD mortality	−8 (−0.43%)	−20 (−0.63%)	−16 (−0.77%)	−13 (−0.61%)
Death from any cause	−13 (−0.14%)	−24 (−0.12%)	−29 (−0.31%)	−23 (−0.19%)

* Assumes a moderate BMI effect of the reduction in SSB consumption: 39% caloric compensation that will result from replacing 1/3 of the reduced SSB consumption with water, 1/3 with diet drinks, and the remaining 1/3 with other caloric beverages such as milk and juice.

** <200% of the Federal Poverty Level.

*** Includes new and recurrent myocardial infarctions.

A reduction in SSB consumption could save California health care treatment costs associated with diabetes and CVD over the decade from 2013–2022. Under a moderate effect on BMI, a 10–20% reduction in SSB intake could lead to $318–$622 million in direct health care costs savings due to prevention of diabetes. An additional $14–$27 million of diabetes-related CHD costs could be avoided. Furthermore, Californians could avoid $550–$1,066 million in CHD treatment costs, overall (Table 5).

**Table 5:** Projected healthcare savings from 2013–2022 after a 10–50% SSB consumption reduction with a moderate BMI effect, in 2012 US dollars – in millions (Percent change).

	Diabetes**	Diabetes-related coronary heartdisease (CHD)***	Total coronary heart disease (CHD)†
10% reduction in SSB consumption*	−$318 (−1.0%)	−$14 (−0.01%)	−$555 (−0.4%)
20% reduction in SSB consumption*	−$622 (−2.0%)	−$27 (−0.03%)	−$1,066 (−0.7%)
50% reduction in SSB consumption*	−$1,480 (−4.7%)	−$66 (−0.07%)	−$2,591 (−1.6%)

* Assumes 39% caloric compensation that will result from replacing 1/3 of the reduced SSB consumption with water, 1/3 with diet drinks, and the remaining 1/3 with other caloric beverages such as milk and juice.

** Diabetes cost is adjusted to only reflect diabetes direct healthcare costs.

*** Diabetes related CHD cost represents excess CHD that could be avoided as a result of the avoided diabetes cases from reduced SSB consumption.

† Reflects total CHD treatment cost.

## Discussion

Reducing SSB consumption could substantially improve health outcomes for all adult Californians and result in considerable cost-savings because of reductions in chronic diseases like diabetes and CVD. The magnitude of the health benefits are projected to be greatest for African Americans, Mexican Americans, and those with limited incomes, populations with the highest rates of diabetes and SSB consumption in California. These findings suggest that reductions in SSB consumption as might be achieved from proposed taxes could have a marked population-wide health benefit for California and have the additional benefit of reducing race/ethnic and income disparities in diabetes and heart disease.

Few studies have examined the range of anticipated health outcomes associated with a reduction in SSB consumption or the impact of a tax as a means to reduce consumption. We previously used a national version of the CVD Policy Model to project the impact of a national excise tax on SSB on health outcomes and costs among U.S. adults and found that such a tax is projected to could prevent 2.4 million diabetes person-years, 95,000 CHD events, 8,000 strokes, and 26,000 premature deaths, while avoiding $17 billion in medical cost from 2010–2020. [14] Several economic studies have examined the impact of taxation of SSB on weight across different income groups, projecting weight loss as a result of these taxes. [38] Economic analyses projecting differences in weight loss by income have yielded differing results. In one analysis, people of limited income were found to be high consumers of SSB and more likely to change their behaviors in order to avoid the tax, but the impact of such changes could blunt weight loss effects because of substitution with generic or bulk products or other items high in sugar particularly in low income populations. [38] A follow-up analysis that considered a range of food items that might be potential substitutes for SSB under taxation failed to find increase in other high sugar items and found instead that even high SSB consumer would be projected to experience reduction in weight as a result of these taxes. [23].

Our study uses a range of assumptions about elasticity and substitution based on these studies and extends these findings by examining additional health outcomes anticipated as a result of lower SSB consumption. Importantly, weight loss is not a primary driver of our results; changes in diabetes and hypertension associated with SSB consumption *independent of weight* contribute the majority of the health benefits we describe. These effects are particularly important among racial and ethnic minority populations and low income populations with high rates of these conditions. Data from CHIS in 2005–2009 among 35–44 year olds show that, on average, African Americans drink 0.51 SSB per day, Mexican Americans 0.59 and in low income groups 0.70 compared with white Californians with 0.47 SSB per day. [17] Racial/ethnic groups have exceptionally high burden of diabetes and obesity in California. In 2007, for adults 18 and over, prevalence rates of diabetes and obesity were 9.2% and 30.1% in Mexican Americans and 11.5% and 35% in African Americans respectively. [39] White Californians, in comparison, had 6.7% prevalence of diabetes and 20.4% of obesity in 2007. [18] Our findings provide a quantitative comparison of the potential health impact of reducing SSB consumption in these subgroups. Whereas 1 in 20,000 Californians would be expected to avoid diabetes over the next decade as a result of this excise tax, the estimates are closer to 3 in 20,000 African Americans, 2 in 20,000 Mexican Americans, and 2 in 20,000 low income Californians.

Controversy has arisen over recent proposals to tax SSB or regulate portion sizes of these beverages with concern that low income and minority communities would be unfairly burdened by these taxes. [40] Our work highlights the proportionately greater health benefits in these communities, an important factor that must also be considered in these discussions. Avoiding chronic illnesses like diabetes and heart disease could result in a variety of health benefits for individuals and economic benefits as well. Although we outline here the healthcare cost-savings that might be experienced from a societal perspective, additional economic benefits to individuals, communities, and society from the reduced disability and premature mortality from avoiding diabetes and heart disease would also be expected. [41] Another potential benefit of taxation for these communities is the proposal to reinvest revenues from these taxes into the communities with the highest rates of chronic diseases for health promoting activities. A recent poll suggests that most Californians would support a tax on SSB if the revenue from such a tax were reinvested in other health-promoting activities in the communities disproportionately affected by diabetes. [42].

The CVD Policy Model on which these California estimates are based is a well-established model that has produced robust projections of the health impacts of changes in risk for cardiovascular disease and has been used to inform health policies for over 25 years. However, all models are limited by the integrity of the inputs for the model. The main effect of SSB consumption on diabetes, blood pressure, and body weight were based on published analyses of observational studies and therefore are subject to unmeasured and residual confounding factors and may not be generalizable to all populations. [11], [12] While we have estimates of physiological effects of lower SSB consumption from several large studies, our estimates of consumer behavior in response to individual and policy-level interventions may differ widely; therefore, we varied the potential reduction of consumption in SSB across a wide range. In addition, the degree to which calories will be substituted for by other caloric foods and beverages, and the impact of a reduction in calories on BMI are also uncertain. We based our estimates on the best available evidence of consumer behavior and energy balance, and to account for this uncertainty, we varied the impact of reduction in consumption of SSB from no effect on BMI to a strong effect on BMI. We used self-reported SSB consumption from CHIS which may be limited by under or over-reporting. We did not account for artificially sweetened beverage consumption; recent studies have found an association between artificially sweetened beverage consumption and increased risk of obesity, type 2 diabetes, metabolic syndrome and CVD [43]; however, the long term health implications are not fully understood. [44] Additionally, our estimates of costs are limited to health care cost; the true societal costs of excess preventable morbidity and mortality include those associated with lost economic productivity from disability and premature mortality from diabetes and CHD. Although some data suggest an effect of SSB consumption on lipid levels, the whether this effect is independent of BMI, therefore we did not include an effect on lipids in our model. [45] This may have underestimated the impact of a reduction on SSB consumption on CHD. Finally, we focused on adults in these projections because the data linking SSB consumption to health outcomes such as diabetes, hypertension, and CHD are available in this age group and are the health outcomes most likely to be observed in high numbers over the duration that we modeled (2013–2022). However, the largest consumers of SSB are adolescents; therefore, the anticipated health impact for California over a longer time horizon is likely to be even greater.

In conclusion, our study projects that the reduction in SSB consumption that is anticipated to result from an excise tax of a penny per ounce could yield substantial population health benefits and cost savings in California, and importantly would result in greater benefits in high-risk populations. Although taxation to curb consumption of SSBs is of considerable interest across the US and globally, [46] the limited adoption of these measures has restricted the types of empirical data on which to base the effect of such policy tools to modify consumer behaviors. The rising tide of diabetes nationally and globally suggests that more effective policy options to curb consumption will continue to be sought and adopted. Whether taxation or other types of regulatory efforts, our study findings suggest that policy strategies capable of effectively reducing SSB consumption may be an important step towards reversing the devastating upward diabetes trends in California and supporting the health of all communities in the state.
